# Beads-Milling of Waste Si Sawdust into High-Performance Nanoflakes for Lithium-Ion Batteries

**DOI:** 10.1038/srep42734

**Published:** 2017-02-20

**Authors:** Takatoshi Kasukabe, Hirotomo Nishihara, Katsuya Kimura, Taketoshi Matsumoto, Hikaru Kobayashi, Makoto Okai, Takashi Kyotani

**Affiliations:** 1 Institute of Multidisciplinary Research for Advanced Materials, Tohoku University, 2-1-1 Katahira, Aoba-ku, Sendai 980-8577, Japan; 2 PRESTO, The Japan Science and Technology Agency (JST), 4-1-8 Honcho, Kawaguchi 332-0012, Japan; 3 The Institute of Scientific and Industrial Research, Osaka University, 8-1 Mihogaoka, Ibaraki, Osaka 567-0047, Japan; 4 Center for Technology Innovation-Materials, Hitachi Ltd., Hitachi, Ibaraki 319-1292, Japan

## Abstract

Nowadays, ca. 176,640 tons/year of silicon (Si) (>4N) is manufactured for Si wafers used for semiconductor industry. The production of the highly pure Si wafers inevitably includes very high-temperature steps at 1400–2000 °C, which is energy-consuming and environmentally unfriendly. Inefficiently, ca. 45–55% of such costly Si is lost simply as sawdust in the cutting process. In this work, we develop a cost-effective way to recycle Si sawdust as a high-performance anode material for lithium-ion batteries. By a beads-milling process, nanoflakes with extremely small thickness (15–17 nm) and large diameter (0.2–1 μm) are obtained. The nanoflake framework is transformed into a high-performance porous structure, named wrinkled structure, through a self-organization induced by lithiation/delithiation cycling. Under capacity restriction up to 1200 mAh g^−1^, the best sample can retain the constant capacity over 800 cycles with a reasonably high coulombic efficiency (98–99.8%).

The world production of silicon (Si) metal in 2014 was about 1,766,400 tons^1^, and about 10% of them is high-purity grade (>4N) for Si wafers which are used for semiconductor industry including integrated circuits and photovoltaic cells. Prior to the production of Si wafers, Si is first prepared by the reduction of SiO_2_ at a very high temperature (>1900 °C) with the presence of reducing agent such as charcoal and coal. The raw Si thus obtained is then reacted with HCl to become SiHCl_3_, which is further rectified into high-purity Si metal. Afterwards, the Si metal is molten at a high temperature above its melting point (>1414 °C), and a large ingot of single-crystalline or polycrystalline Si is prepared typically by the Czochralski or floating zone technique with taking more than several tens of hours. Thus, the production of Si ingots is extremely energy-consuming, *i.e.*, environmentally unfriendly, and such a high-temperature process inevitably increases the price of Si. Then, the ingots are finally cut into Si wafers and they are used for a variety of applications. What is awfully inefficient is the fact that ca. 45–55% of the high-quality Si is lost simply as sawdust in the cutting process^2^. This means that a large amount of energy used for the production of the Si ingots is idly abandoned. Hence, the reuse of the Si sawdust is highly desired from the sustainable point of view. One of the potential applications of the Si sawdust could be a high-capacity anode material for lithium-ion batteries (LIBs). Si has a very high theoretical capacity (3572 mAh g^−1^)^3^ compared to that of conventional graphite (372 mAh g^−1^), and there have been a great deal of researches reporting the development of Si-based high-capacity materials (800–3000 mAh g^−1^) for the LIB application as found in comprehensive review papers^4^,^5^. In 2015, the world demand for graphite in all batteries is estimated to be 125,000 tons^6^. If graphite is replaced by Si, the necessary amount of Si is estimated to be ca. 15,000–58,000 tons, considering its high capacity. Accordingly, the amount of Si sawdust (ca. 88,320 tons) meets the demand of anode materials for LIBs.

However, there are several technical hurdles to realize the reuse of Si sawdust for LIBs. First, Si sawdust is obtained as sludge mixture containing other contaminants, and therefore, a purification process is necessary. For the manufacture of Si wafers, a Si ingot is sliced by a wire saw, and during this cutting process, a large amount of coolant is used and thus it remains in the sawdust. At the same time, metal impurities derived from the wire saw are mixed in the sawdust. Additionally, the cutting process usually needs to use a substrate such as graphite, alumina, and polymers, and sawdust of the substrate is also mixed. Hence, these impurities should be removed prior to use for LIBs. In this work, we recover Si sawdust whose quality is high enough for LIB application, based on our proposed technique on the production of Si nanoparticles from the Si sawdust^7^. The second problem is about how to mold Si sawdust into an appropriate nanostructure which is the key factor to achieve a high performance and a long-term durability as an anode for LIBs. Si has intrinsic problems of (i) low conductivity, (ii) low reaction rate with lithium, and (iii) large volume change upon lithiation/delithiation. To overcome these problems, it is generally anticipated that the following means are of importance: (I) the domain size of Si should be less than a few hundred nanometers, (II) Si is necessarily mixed with conductive additives, and (III) buffer nanospace should be placed around Si^8^. Thus far, high performance has been achieved in a variety of Si-based nanomaterials such as Si nanowires^9^,^10^,^11^, Si nanotubes^12^,^13^, porous Si particles^14^, carbon inverse-opal decorated with Si nanolayer/nanoparticles^15^,^16^, C/Si or Si/C core-shell nanowires^11^,^17^. However, most of them are produced by multistep, costly, and environmentally unfriendly processes. In addition, it is impossible to mold Si sawdust into either of these specific nanostructures. Thus, Si nanoparticles have been prepared from Si sawdust for LIBs, by means of plasma jet treatment^18^, ultrasonic spray-draying^19^, and high energy ball milling^20^. They show high capacity (1000–2000 mAh g^−1^) at early cycles, but the capacity is faded by 50–150 cycles^18^,^19^,^20^, and the cyclability should be improved much more for practical application. The reason for the insufficient cyclability could be the morphology of the materials prepared from Si sawdust, *i.e.*, discrete Si nanoparticles. One can be aware that most of the afore-mentioned high-performance Si-based materials possess a continuous Si framework like fibers in contrast to isolated and discrete Si particles. This is because such continuous Si frameworks show specific structure change during charge/discharge cycling^21^,^22^,^23^,^24^,^25^. By repeating volume expansion by lithiation and contraction by delithiation, the continuous Si frameworks turn into porous framework resembling wrinkled papers, in which a high performance can be retained. We have indeed demonstrated that Si nanoparticles which are tightly connected each other to have a continuous structure can be readily changed to high-performance ‘wrinkled’ Si framework upon the repetition of lithiation/delithiation^21^,^22^. Later on, almost the same phenomenon has been reported also by Park *et al*.^23^. Moreover, we have reported the detailed structure of the wrinkled Si framework to elucidate its high performance^21^. The formation of similar structures to the wrinkled structure have been reported in the Si nanowires^24^ and carbon nanotube covered by Si nanolayer^25^, and also in Ge^26^,^27^,^28^,^29^,^30^,^31^,^32^,^33^,^34^ and SnO_2_^35^, which involve large volume expansion/contraction upon lithiation/delithiation. In these examples, the presence of a continuous framework consisting of a nano-sized active material (Si, Ge, or SnO_2_) is the key factor for the formation of the wrinkled structure, thereby exhibiting a high performance. By contrast, discrete Si nanoparticles prepared by the practical milling process never show such structure transformation, and their performance is quite low as a result^22^. Thus, it is a challenge to realize the wrinkled structure in discrete Si nanoparticles prepared by milling process which is practically sound.

In this work, we demonstrate that an appropriate milling process can convert Si sawdust into nanoflakes having a very thin thickness (ca. 16 nm) and a large domain size (0.2–1 μm), which functions as a continuous framework. As a result, the nanoflakes are transformed into the wrinkled structure during charge/discharge cycling by self-organization, and exhibit better performance than discrete and spherical Si nanoparticles. Moreover, we have examined the effect of carbon-coating over the Si nanoflakes, and also several different cell-assembling methods according to literature, to further enhance the performance of the Si nanoflakes. The obtained results demonstrate an excellent feasibility of the recycling of Si sawdust as a high-performance anode material for LIBs.

## Results and Discussion

### Preparation and Characterization of Si nanoparticles

Si sawdust (Si(sd); about 0.2–4 μm in particle size) was produced from real Si-sawdust sludge derived from phosphorus-doped n-type crystalline Si ingots with a resistivity of 1–2 Ω cm. Shortly, Si(sd) was prepared by washing the waste sludge with acetone to remove the coolant. Si(sd) thus obtained contains 4 wt% of graphite sawdust which is derived from a graphite substrate used for the cutting process of the Si ingots. Fortunately, graphite functions as an anode of LIBs and it is not necessary to be removed. For comparison, commercial Si powder (Si(com); purity 99.9%, average particle size 1–5 μm, Alfa Aesar) was used. Each of the Si powdery samples was milled with the presence of water or isopropanol down to nanoscale by the following two methods: (1) ball-milling with a Pulverisette 7 premium line apparatus (Fritsch Co., Ltd.)^22^, and (2) beads-milling with a LMZ015 apparatus (Ashizawa Fintech Ltd.). Then the sample was washed with 1 wt% HF solution to remove a surface SiO_2_ layer. The more detail information is shown in Supplementary Information. The Si nanoparticles thus obtained are referred to as Si(X)-Y(Z) where X expresses the raw Si powder (sd or com, corresponding to sawdust and commercial Si powders, respectively), Y stands for the type of the milling apparatus (ba or be, corresponding to ball-milling and beads-milling, respectively), and Z stands for the solvent (w or ipa, corresponding to water and isopropanol, respectively).

**Figure 1:**
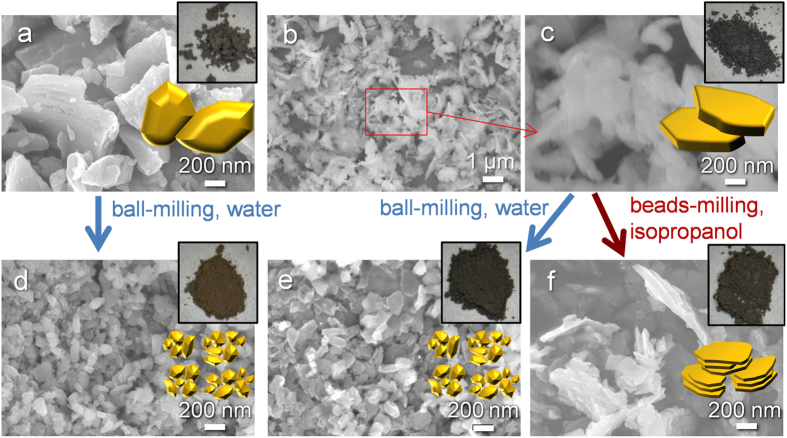
Photographs, SEM images, and symbolic illustrations of (**a**) Si(com), (**b,c**) Si(sd), (**d**) Si(com)-ba(w), (**e**) Si(sd)-ba(w), and (**f**) Si(sd)-be(ipa). The photographs and the symbolic illustrations are shown as upper and lower insets, respectively, in (**a,c–f**).

Figure 1a–f show photographs and scanning electron microscopy (SEM) images of the Si samples, together with their symbolic illustrations. Si(com) (Fig. 1a) is crushed particles with a diameter of 1–5 μm. By contrast, Si(sd) (Fig. 1b and c) has a flaky shape since it is produced by a wire saw, and this process results in the inclusion of graphite impurity (4.0 wt%). As described above, graphite is the conventional anode material for LIBs, and therefore, the presence of such a small amount of graphite does not seriously lower the performance of the Si materials. The elemental mapping of Si(sd) revealed a homogeneous distribution of carbon in this sample (Supplementary Fig. S1a–c), and we cannot distinguish graphite particles from the Si particles, suggesting that graphite exists as a very fine powder. Despite the very different morphologies between Si(com) and Si(sd), their milled products, Si(com)-ba(w) (Fig. 1d) and Si(sd)-ba(w) (Fig. 1e), have almost the same shape, *i.e.*, spherical particles with a diameter of less than 200 nm. Thus, the initial Si shape does not affect the resulting morphology of this ball milling process using water. On the other hand, the beads-milling process with the presence of isopropanol yields nanoflake morphology as is found in Si(sd)-be(ipa) (Fig. 1f) and also in Si(com)-be(ipa) (Supplementary Fig. S2a). These findings indicate that the particle shape of the precursor does not affect the final product shape in both milling processes. To examine the sole effect of a liquid medium, the ball-milling process was applied to Si(com) with the presence of isopropanol instead of water. The resulting sample, Si(com)-ba(ipa) has intermediate morphology between nanosphere (Fig. 1d,e) and nanoflake (Fig. 1f and Supplementary Fig. S2a), as is found in Supplementary Fig. S2b. Thus, isopropanol makes the resulting morphology more like flake. Considering the fact that there is no great difference between viscosities of isopropanol (1.77 mPa·s at 30 °C) and water (0.78 mPa·s at 30 °C), the effect of the liquid media is ascribed to their different chemical properties rather than the rheological properties. Single Si crystal has several cleavage planes such as (111), (100), and (110), in which the crystal tends to be smoothly cut. Si(sd) is derived from a single crystal ingot, and therefore, it is no wonder that Si(sd) is crushed into flake-like shape rather than spherical. This could be the case of isopropanol. On the other hand, in the case of water, it intensively and immediately oxidize fresh Si surfaces that are generated by milling. Indeed, when ball-milled Si(sd) with water was treated by HF to remove the surface SiO_2_ layer, the original sample weight was decreased down to approximately 35%. The weight loss occurs not only by SiO_2_ removal but also by technical issues such as filtration, but in the case of isopropanol, the same HF treatment (including the same filtration process) achieves a recovery of 64%. These results clearly indicate that water tends to oxidize Si during the milling process much more than isopropanol, which generally works as a reducing agent. Thus, the intense surface oxidation by water may hinder cleavage along the crystal planes, resulting in the formation of spherical nanoparticles. Moreover, the type of the milling apparatus is also a key factor. The beads-milling apparatus used in this work enables to apply greater shear stress than the case of the ball-milling apparatus.

The color change of the samples is worth noting. As is found from their photographs, both Si(com) and Si(sd) are dark grey powder (Fig. 1a,c). By the ball-milling treatment on Si(com), its color turned to be brown (Fig. 1d), which is generally seen in Si nanoparticles^8^. Though the particle size of Si(sd)-ba(w) is almost the same as that of Si(com)-ba(w), the color of the former is dark grey (Fig. 1e). This is owing to the presence of graphite in this sample (4.8 wt%). It is noteworthy that a simple mixing of Si(com)-ba(w) with 5.0 wt% of graphite powder with a motor does not change the brown color at all (Supplementary Fig. S3). The dark grey color of Si(sd)-ba(w) thus suggests a homogeneous dispersion of the graphite. Indeed, the elemental mapping with energy dispersive X-ray spectrometer (EDX) proved the very uniform distribution of carbon in this sample (Supplementary Fig. S1d–f). Similarly, Si(sd)-be(ipa) has also grey color (Fig. 1f) and 5.4 wt% of graphite is uniformly distributed also in this sample (Supplementary Fig. S1g–i).

**Figure 2:**
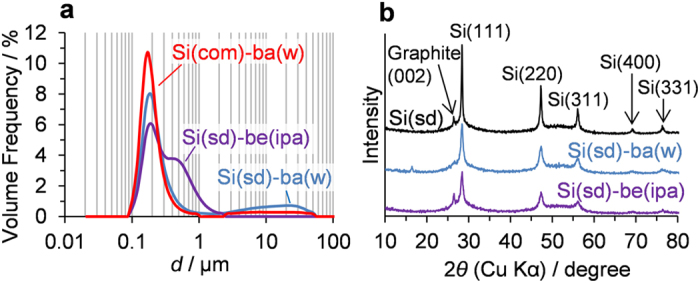
(**a**) Particle-size distributions of the milled samples measured by the laser diffraction technique. (**b**) XRD patterns of Si(sd) and its milled samples.

The specific surface areas of Si(com)-ba(w), Si(sd)-ba(w), and Si(sd)-be(ipa) are 57, 58, and 59 m^2^ g^−1^, respectively. By the assumption of spherical particle shape, their average particle sizes can be calculated as 45, 44, and 44 nm. The former two values almost agree with the sizes observed in their SEM images (Fig. 1d,e), since they have spherical morphology. On the other hand, the nanoflake diameter of Si(sd)-be(ipa) is estimated to be about 0.2–1 μm from its SEM image (Fig. 1f). With the specific surface area and the nanoflake diameter, the thickness of the nanoflake is therefore estimated as 15–17 nm, which is extremely thin. The actual particle-size distributions of these samples were analysed by a laser diffraction technique, as shown in Fig. 2a. Generally, nanoparticles are likely to aggregate and form secondary particles. Si(com)-ba(w) and Si(sd)-ba(w) thus have a major peak around 0.1–0.3 μm, corresponding to the secondary particle sizes. On the other hand, Si(sd)-be(ipa) has a major peak around 0.1–0.3 μm with a shoulder around 0.3–1.0 μm. Since Si(sd)-be(ipa) has the nanoflake morphology, each of nanoflakes should be easily stacked to form thicker secondary flakes, while the stacking does not increase the diameter of the flakes. Indeed, the range of the shoulder well accords to the diameter of the sample observed by SEM (Fig. 1f). Therefore, the major peak and the shoulder can be ascribed to the thickness and the diameter of the stacked nanoflakes, respectively. Thus, Si(com)-ba(w) and Si(sd)-ba(w) have the secondary particle size of ca. 0.1–0.3 μm by weak physical aggregation of spherical nanoparticles, while Si(sd)-be(ipa) has a similar dimension as a continuous framework of nanoflake. As shown later, the latter exhibits better performance.

XRD patterns of Si(sd) and its milled samples are shown in Fig. 2b. Si(sd) shows sharp peaks of Si, together with weak and broad peaks around 25–35° and 45–60°, which are not observed in Si(com)^22^. The broad peaks suggest the presence of very small crystalline Si in Si(sd). Additionally, there is also a peak of graphite (002), but no other crystalline impurities are detected. Thus, the present washing process for the raw Si-sawdust sludge successfully removes inorganic impurities except for graphite. In the milled samples, the sharp peaks of Si become much broader, which accords to the very small primary particle sizes of these samples estimated from the SEM images and the specific surface areas. The crystallite size was calculated for each of the Si peaks by the Scherrer equation, and the result is shown in Supplementary Table S1. The crystallite size of Si(sd)-be(ipa) is in the range of 10–30 nm, and this is much smaller than the nanoflake diameter which is estimated by specific surface area and SEM. Thus, the nanoflake is found to be a polycrystalline solid. In Si(sd)-ba(w), the graphite (002) peak is almost lost, indicating the breaking down of the graphite crystallite. On the other hand, Si(sd)-be(ipa) retains the sharp peak of graphite. As shown before, the beads milling with isopropanol yields flaky Si particles, and therefore, flaky graphite would not be broken down by this type of milling. The presence of the well-dispersed graphite (Supplementary Fig. S1i) is advantageous to achieve a lower inner resistance for LIB application.

### Charge/discharge performance of Si nanoparticles

**Figure 3:**
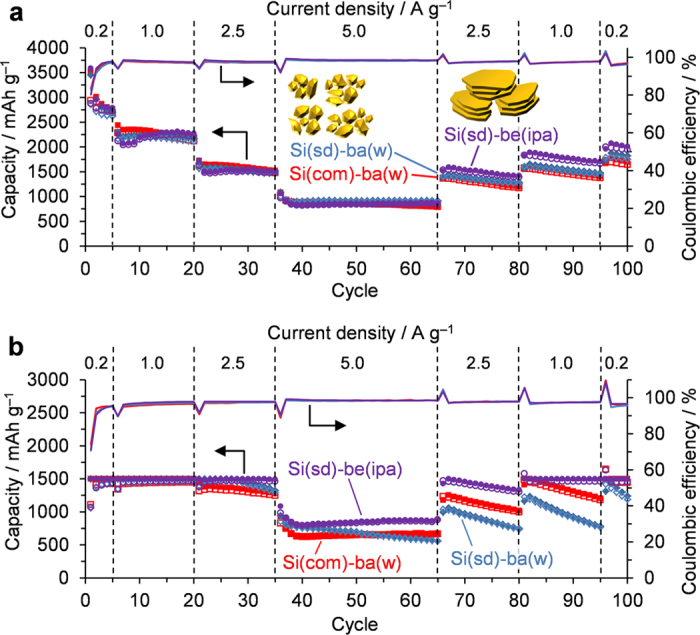
Charge/discharge capacities (open/solid symbols) and coulombic efficiency (solid lines) vs. cycle number. (**a**) Without and (**b**) with discharge-capacity restriction up to 1500 mAh g^−1^ measured in 1 M LiPF_6_ in a mixture of ethylene carbonate and diethyl carbonate (1:1 by volume).

**Figure 4:**
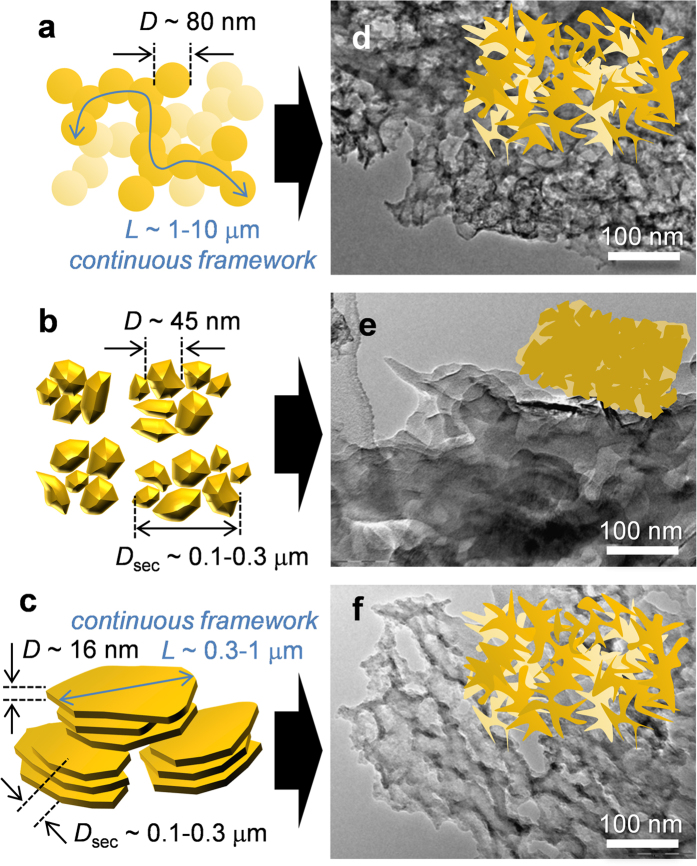
The relationship between the initial structures of Si (**a–c**) and the corresponding ones after 100 charge/discharge cycles (**d–f**) under a capacity restriction of 1500 mAh g^−1^. In (**a–c**), *D, L*, and *D*_sec_ are particle diameter (**a,b**) or flake thickness (**c**), a length of a continuous structure, and the size of secondary aggregation estimated by the laser diffraction, respectively. (**d–f**) are TEM images of Si(CVD), Si(sd)-ba(w), and Si(sd)-be(ipa), respectively, after 100 charge/discharge cycles. Insets represent their structures: wrinkled structure (**d,f**) and aggregated lump (**e**).

Charge/discharge performance of Si nanoparticles were examined by a 2032-type coin cell. A counter electrode was Li foil, and an electrolyte was 1 M LiPF_6_ in a mixture of ethylene carbonate and diethyl carbonate (1:1 by volume ratio). See “Method I” in the experimental section as for the details. Figure 3 shows charge/discharge (delithiation/lithiation) capacities and coulombic efficiencies of Si nanoparticles during 100 cycles without and with capacity restriction (see Supplementary Fig. S4a–c for the original charge/discharge curves of the samples). All of the curves are basically similar to those in the typical Si-nanoparticle materials: lithiation occurs at 0–0.3 V, and delithiation at 0.2–0.6 V^14^,^21^,^22^,^36^. Si(sd)-be(ipa) exhibits superior cyclability and rate capability to the other two samples both in Fig. 3a,b. This could be ascribed to the presence of the highly dispersed flaky graphite and/or the nanoflake morphology of Si. To examine which factor is important, a counterpart sample which has the same nanoflake morphology but does not contain graphite was prepared by the beads-milling of Si(com) (Si(com)-be(ipa) in Supplementary Fig. S2a). The charge/discharge performance of Si(com)-be(ipa) is shown in Supplementary Fig. S5, which exhibits good performance similar to that of Si(sd)-be(ipa) even without graphite, indicating that the nanoflake morphology is more significant to achieve a high performance. The performance of Si(com)-be(ipa) is comparable to that of CVD-derived Si (Si(CVD))^21^. The morphologies of Si(CVD), Si(sd)-ba(w), and Si(sd)-be(ipa), are illustrated in Fig. 4a–c, respectively. They are carbon-black-like networking nanoparticles (Fig. 4a), discrete and spherical nanoparticles (Fig. 4b), and nanoflakes (Fig. 4c). Si(CVD) consists of nanoparticles with the diameter of ca. 80 nm, and the nanoparticles are tightly connected to form a continuous network structure (length is ca. 1–10 μm)^21^,^22^. Such a structure, *i.e.*, nanosized framework with a long continuous structure, has been proved to give better performance than that of discrete nanoparticles shown in Fig. 4b 22. As found in Fig. 4c, Si(sd)-be(ipa) has a continuous framework whose dimension is comparable to Si(CVD), and the high-performance of the former material shown in Fig. 3 can be thus rationally understood. On the other hand, weak physical aggregation found in the discrete nanoparticles (Fig. 4b) is not effective to enhance the performance of Si.

Even not being critical, the presence of highly dispersed flaky graphite in Si(sd)-be(ipa) improves its cyclability, as is found its better capacity retention after the 80th cycle than Si(com)-be(ipa) (see Supplementary Fig. S5b). Si(com)-ba(w), Si(sd)-ba(w), and Si(sd)-be(ipa) exhibit relatively high coulombic efficiencies of 84, 83, and 80%, respectively, in the first cycle in Fig. 3a, and all of them quickly achieve over 96% after the 4^th^ cycle.

### The formation of wrinkled structure

We have revealed that the structural change of Si nanoparticles during long-term charge/discharge cycles strongly depends on the particle connectivity^21^,^22^. As shown in Fig. 4a,d, Si(CVD) turned into a high-performance wrinkled structure, whereas discrete Si nanoparticles simply transformed into a low-performance aggregated lump (Fig. 4b,e)^21^,^22^. The wrinkled structure can exhibit good cyclability and rate capability, because this structure has a considerably low inner resistance and contains nanopores which play a role of buffer of Si expansion^21^. As mentioned in the introduction, nano-sized Si having a continuous framework tends to transform into the high-performance wrinkled structure by repeating volume expansion/contraction induced by lithiation/delithiation. We found that Si(sd)-be(ipa) also transforms into the wrinkled structure as shown in Fig. 4f. Starting from completely different initial shapes (Fig. 4a,c), both Si(CVD) and Si(sd)-be(ipa) eventually turned into the similar wrinkled structures (Fig. 4d,f). To the best of our knowledge, this is the first report of the formation of the wrinkled structure in discrete Si nanoparticles prepared by a practical milling process, and this is the reason for the high-performance of this sample.

**Figure 5:**
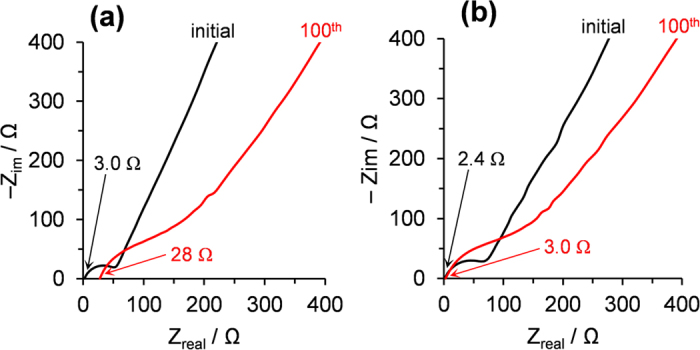
Nyquist plots of (**a**) Si(sd)-ba(w) and (**b**) Si(sd)-be(ipa) before the charge/discharge cycles and those after 100th cycles under capacity restriction up to 1500 mAh g^−1^. Ohmic resistance of a half cell is described for each of the Nyquist plots.

To obtain further insight of the wrinkled structure formed from Si(sd)-be(ipa) and to clarify its advantage over the aggregated lump shown in Fig. 4e, electrochemical impedance spectroscopy was applied to Si(sd)-ba(w) (Fig. 4b) and Si(sd)-be(ipa) (Fig. 4c). Figure 5 shows Nyquist plots of these samples before charge/discharge cycles and those after the 100th cycles under capacity restriction up to 1500 mAh g^−1^. Note that the charge/discharge cycles in this measurement corresponds to those shown in Fig. 3a, and the structures before and after the 100th cycles correspond to Fig. 4b,c,e,f. The minimum Z_real_ value at a high frequency (100 kHz) represents the ohmic resistance of a half cell, and this includes contact resistance, inner resistance of the electrodes, and electrolyte resistance. The region of a semi-circle or broken semi-circle (100 to 0.1 kHz) is generally associated with interfacial phenomena, either the charge transfer or the formation of solid-electrolyte interface (SEI). In both samples, the initial semi-circles become loosely broken ones after the 100th cycle, indicating the formation of SEI. The critical difference can be seen in the ohmic resistance after the 100th cycle. While in Si(sd)-ba(w) having the aggregated lump (Fig. 4e), resistance became very large (28 Ω), the wrinkled structure formed from Si(sd)-be(ipa) (Fig. 4f) retained a small resistance of 3.0 Ω. This is ascribed to the presence of a good conductive network in the wrinkled structure. Thus, simply by using a practical milling method, high-performance Si anode can be prepared.

### The effect of carbon coating

**Figure 6:**
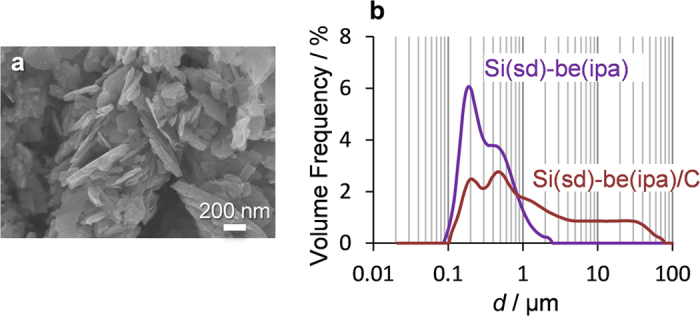
(**a**) SEM image of Si(sd)-be(ipa)/C and (**b**) particle-size distributions of Si(sd)-be(ipa) and Si(sd)-be(ipa)/C measured by the laser diffraction technique.

We then examined the effect of carbon-coating on Si(sd)-be(ipa). Si(sd)-be(ipa) was covered with a carbon layer by chemical vapor deposition (CVD) (see details in Supplementary information). The carbon-coated Si(sd)-be(ipa) thus obtained is referred to as Si(sd)-be(ipa)/C. The weight fraction of carbon in the carbon-coated sample, Si(sd)-be(ipa)/C, is determined to be 13 wt% by the elemental analysis. The SEM image of Si(sd)-be(ipa)/C (Fig. 6a) reveals that its morphology is almost the same as that of Si(sd)-be(ipa) (Fig. 1f) even after the carbon coating. This suggests that Si(sd)-be(ipa) is uniformly covered with a thin carbon layer. Particle size distributions of Si(sd)-be(ipa) and Si(sd)-be(ipa)/C are shown in Fig. 6b. By the carbon coating, the intensity of the original peak in Si(sd)-be(ipa) is decreased, and the distribution is expanded to several tens micron meters. Thus, it is clear that the carbon coating interconnects the stacked Si nanoflakes of Si(sd)-be(ipa) to form a larger continuous structure.

**Figure 7:**
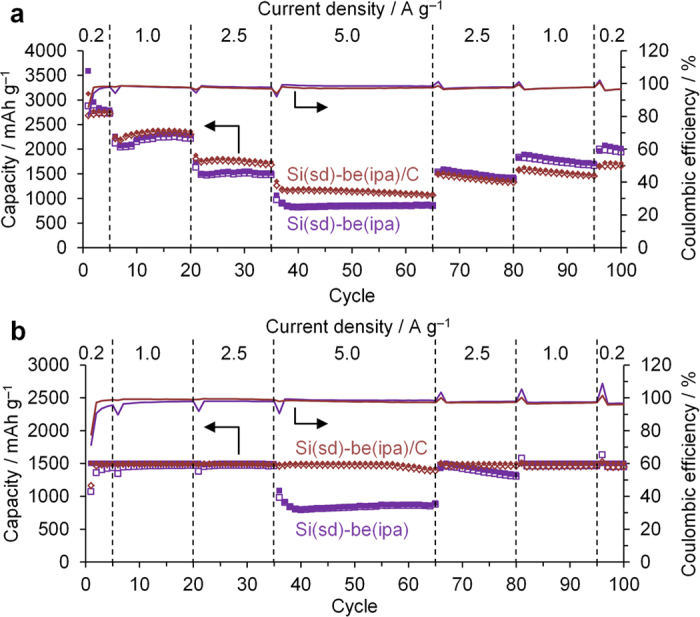
Charge/discharge capacities (open/solid symbols) and coulombic efficiency (solid lines) vs. cycle number (**a**) without and (**b**) with discharge-capacity restriction up to 1500 mAh g^−1^ measured in 1 M LiPF_6_ in a mixture of ethylene carbonate and diethyl carbonate (1:1 by volume).

The charge/discharge capacity and the coulombic efficiency of Si(sd)-be(ipa)/C are shown in Fig. 7, together with that of the uncoated sample (Si(sd)-be(ipa)). The original charge/discharge curves of the carbon-coated sample are shown in Supplementary Fig. S4d. The 1st discharge (lithiation) capacity of Si(sd)-be(ipa)/C is 3126 mAh g^−1^, which is lower than that (3587 mAh g^−1^) of Si(sd)-be(ipa), due to the presence of 13 wt% of carbon. On the other hand, the 1st coulombic efficiency is slightly improved by the carbon coating, from 80% to 85%. It is noteworthy that the carbon-coated sample exhibits a better rate capability at 5.0 A g^−1^ both in Fig. 7a,b. Thus, the larger continuous structure formed by the carbon coating further improves the rate capability of Si(sd)-be(ipa). In Si(sd)-be(ipa)/C, it achieves 1500 mAh g^−1^ at 5 A g^−1^ only under the capacity restriction. This is because the structure change is affected by the capacity restriction. Under capacity restriction, structure change proceeds more slowly, and a high performance could be retained in this case. We have reported the similar results in Si(CVD)^21^. However, the effect of the capacity restriction on the structure transition highly depends on samples^22^. Hence, there are many cases in which the capacity under capacity restriction is lower than that without restriction^22^.

### Comparison of cell-assembly methods

**Figure 8:**
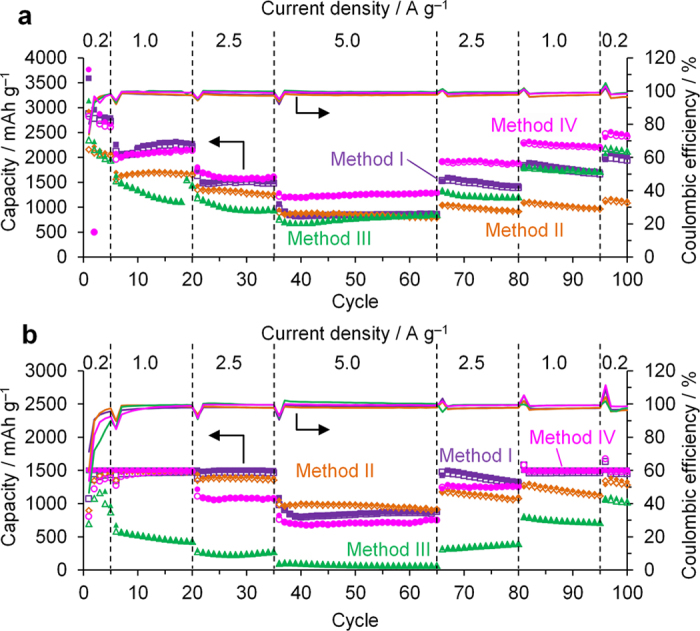
Comparison of four different cell-assembly methods on the charge/discharge capacities (open/solid symbols) and coulombic efficiency (solid lines) of Si(sd)-be(ipa). (**a**) Without and (**b**) with discharge-capacity restriction up to 1500 mAh g^−1^.

It is well known that binder polymers and electrolyte additives^37^,^38^,^39^,^40^,^41^ greatly affect the performance of Si-based anodes. To examine their effects on the Si nanoparticles made from Si(sd), four different cell-assembly methods were selected from literature, which have achieved a high performance in Si anodes. See details about the four methods (Method I, II, III, and IV) in the experimental section. The electrode densities of the Method I, II, III, and IV are 0.2–0.3, 0.3–0.4, 0.2, and 0.2–0.4 g cm^−3^, respectively. Figure 8 shows charge/discharge capacities and coulombic efficiencies of Si(sd)-be(ipa) measured by using the four different methods. Both in Fig. 8a,b, Method IV shows the best capacity retention at the end of the cycles. In addition, the rate capability is also good, as found from its high capacity retention at 5.0 A g^−1^.

Delpuech *et al*. have reported that esterification reaction between –COOH groups in CMC and Si–OH groups on the Si surface is enhanced in pH 3 buffered solution^36^. As a result, Si is tightly fixed to the electrode which can sustain the volume change during charge/discharge cycling. To Si(sd)-be(ipa), this mechanism seems to work very well. As for Method II, it has been reported that sodium poly(acrylic acid) very well covers the surface of Si and more increases the mechanical strength and adhesive strength than poly(vinylidene fluoride) and sodium CMC^42^. However, the present results suggest that the use of the CMC binder together with pH control is even better for the present Si nanoflake than sodium poly(acrylic acid), probably due to the presence of the rigid ester bonds. The pH of a sodium poly(acrylic acid) solution is 6–7^43^, and the –COONa groups of the polymer seem not to take place the esterification reaction with Si–OH groups. As for Method III, polyimide is very hard and can strongly fix Si particles in its matrix^44^. However, such a hard polymer has also a brittle aspect, and crucks can be formed upon a large volume change of Si (up to 4 times larger than its original volume)^8^ by lithiation. Once crucks are formed, such a hard polymer which is poor in elasticity cannot retain Si particles. In Method I, SBR plays a role of elastic binder which can be largely deformed and retain Si even upon its large volume expansion/contraction. However, there is no chemical bonding between Si and binder polymers in Method I. Thus, Method IV is found to be the best way to achieve a good cyclability together with a good rate capability.

### Long-term cyclability of Si(sd)-be(ipa)/C

**Figure 9:**
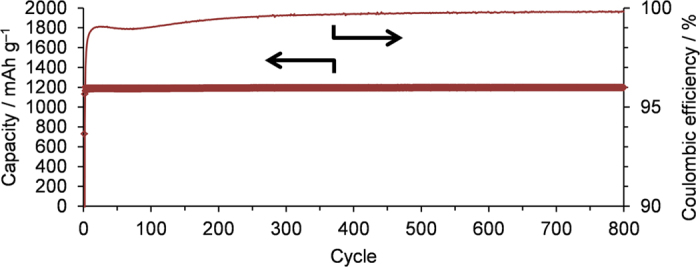
Charge/discharge capacities (open/solid symbols) and coulombic efficiency (solid lines) vs. cycle number under discharge-capacity restriction up to 1200 mAh g^−1^ measured by using Method IV. A current density is 960 mA g^−1^.

We have finally examined a long-term cyclability of the best sample (Si(sd)-be(ipa)/C) by using the best cell-assembly method (Method IV), as shown in Fig. 9. Under the restriction of the lithiation capacity up to 1200 mAh g^−1^, the resulting test cell can retain a constant capacity over 800 cycles. Moreover, the coulombic efficiency during the cycling is as high as 99.0–99.8% except for the initial 4 cycles. For practical application, very high capacity (>1200 mAh g^−1^) is actually not necessary because of the capacity limitation in a cathode side^3^. What is more important is cyclability and coulombic efficiency. Figure 9 evidently proves a promising performance of Si(sd)-be(ipa)/C in terms of these criteria.

## Conclusions

We have successfully demonstrated a way of recycling waste sludge of Si sawdust into a high-performance anode material for lithium-ion batteries. By using a practical beads-milling apparatus and a green solvent, isopropanol, the Si sawdust can be converted into high-performance Si nanoflakes. Moreover, the performance can be further enhanced by carbon-coating, capacity restriction, and an appropriate cell-assembling method. Under capacity restriction up to 1200 mAh g^−1^, the best sample can retain the constant capacity over 800 cycles with a reasonably high coulombic efficiency (99–99.8%).

## Methods

### Characterization

Morphology of the samples was observed by a scanning electron microscope (SEM: S-4800, Hitachi, Ltd.) and a transmission electron microscope (TEM: JEM-2010, JEOL Ltd.). Elemental mapping of the samples was carried out by an energy dispersive X-ray spectrometer (EDX; EDAX inc., Genesis XM2M) attached to the SEM instrument. The particle-size distribution of the sample powder was measured by a laser diffraction technique (MT3000/S3500, MicrotracBEL Corp.). The crystallite size of Si was estimated by X-ray diffraction (XRD: XRD-6100, Shimadzu). The amounts of carbon in Si(sd), Si(sd)-ba(w), Si(sd)-be(ipa) and Si(sd)-be(ipa)/C were determined by using an elemental analyzer (micro corder JM10 Elemental Analyzer, J-Science Lab Co., Ltd.).

### Assembling coin cells

In this work, the following four methods were used for the assembly of a 2032-type coin cell containing a Li foil (counter electrode) and a polypropylene separator in an Ar-filled glove box. In each of the methods, a working electrode is circular shape with a diameter of 16 mm.

Method I^21^,^22^: The sample (active material) is mixed with conductive additive (Denka black (DB), Denki Kagaku Kogyo Co., Ltd.) and binder polymers (carboxymethylcellulose (CMC), (DN-10L, Daicel Fine Chem Ltd.); and styrene butadiene rubber (SBR) (TRD2001, JSR Corporation) dissolved in water) to prepare a slurry. The weight ratio is sample:DB:CMC:SBR = 67:11:13:9. The resulting slurry was pasted on a copper foil. After dried up, the foil was cut out to be a working electrode. The electrolyte was 1 M LiPF_6_ in a mixture of ethylene carbonate and diethyl carbonate (1:1 by volume ratio). In this work, Method I is basically applied unless otherwise noted.

Method II^43^: Sodium poly(acrylic acid) was used as a binder. The weight ratio is sample:DB:binder = 80:10:10. The electrolyte was the same as that of Method I.

Method III^45^: Polyimide was used as a binder. The weight ratio is sample:DB:binder = 75:10:15. In the aforementioned electrolyte solution, 2 wt% of vinylene carbonate and 10 wt% of fluoroethylene carbonate were added.

Method IV^36^,^46^,^47^: An active material, conductive carbon black (SUPER C65, TIMCAL Ltd.) and carboxymethyl cellulose (DS = 0.7, *M*_*w*_ = 90,000) were mixed with a buffer solution of pH = 3 (KOH + citric acid) by using a mortar and then a planetary mixer to prepare a homogeneous slurry. After the slurry was pasted on a copper foil, it was dried at 80 °C for 1 h under air, and at 100 °C for 2 h under vacuum. Then, the foil was cut into circular shape to prepare a working electrode. The weight ratio of the active material, the conductive additive, and CMC was 80:12:8. The electrolyte is the same as that of Method III.

The details about the four methods can be found in Supplementary information.

### Electrochemical measurement

To obtain Li insertion/extraction capacities, the coin cell was galvanostatically charged/discharged between 0.01 and 1.5 V versus Li/Li^+^ for 100 cycles by using a battery charge/discharge unit (Hokuto Denko Co., Tokyo, Japan;HJ1001) at 25 °C. Current density was changed from 0.2 to 5 A g^−1^ during the cycling. Additionally, a similar 100 cycle run was performed under the capacity restriction for Li insertion up to 1500 mAh g^−1^. For Si(sd)-ba(w) and Si(sd)-be(ipa), electrochemical impedance spectrum (EIS) during the cycling was examined after the 1, 5, 20, and 100 cycles. After delithiation up to 1.5 V, and keeping the potential for 60 min, the EIS measurements were conducted at 1.5 V with an amplitude of 5 mV in the frequency range from 100 kHz to 10 mHz. Some of the samples were taken out from the coin cells after charge/discharge cycling, and directly observed with TEM. For a coin cell assembled by Method IV using Si(sd)-be(ipa)/C, a long term cycling run was performed under almost the same conditions as reported by Gauthier *et al*.,^46^,^47^ *i.e.*, galvanostatic charge/discharge cycling between 0.005 V and 1.0 V at 25 °C with a current density of 960 mA g^−1^.

## Additional Information

**How to cite this article:** Kasukabe, T. *et al*. Beads-Milling of Waste Si Sawdust into High-Performance Nanoflakes for Lithium-Ion Batteries. *Sci. Rep.* **7**, 42734; doi: 10.1038/srep42734 (2017).

**Publisher's note:** Springer Nature remains neutral with regard to jurisdictional claims in published maps and institutional affiliations.

## Supplementary Material

Supplementary Information
