# Supplementary material for: Beads-Milling of Waste Si Sawdust into High-Performance Nanoflakes for Lithium-Ion Batteries
Source: Sci Rep. 2017 Feb 20;7:42734. doi: 10.1038/srep42734 (PMC5316999; doi:10.1038/srep42734)
Supplement: Supplementary Information [file srep42734-s1.pdf]

Supplementary Information

Beads-Milling of Waste Si Sawdust into High-Performance Nanoflakes for Lithium-Ion Batteries

Takatoshi Kasukabe[1], Hirotomo Nishihara[1,2*], Katsuya Kimura[3], Taketoshi Matsumoto[3], Hikaru Kobayashi[3], Makoto Okai[4] & Takashi Kyotani[1]

[1] Institute of Multidisciplinary Research for Advanced Materials, Tohoku University, 2-1-1 Katahira, Aoba-ku, Sendai 980-8577, Japan

[2] PRESTO, The Japan Science and Technology Agency (JST), 4-1-8 Honcho, Kawaguchi 332-0012, Japan

[3] The Institute of Scientific and Industrial Research, Osaka University, 8-1 Mihogaoka, Ibaraki, Osaka 567-0047, Japan

[4] Center for Technology Innovation-Materials, Hitachi Ltd., Hitachi, Ibaraki 319-1292, Japan

[*] E-mail: nisihara@tagen.tohoku.ac.jp

S1. Experimental details

Milling process

(1) Ball-milling

Each of the powdery Si samples was first put into a closed vessel whose inner surface is zirconia, together with zirconia beads (*d* = 0.1 mm) and a solvent (pure water or isopropanol). After the closed vessel was filled with Ar, a high-power planetary ball-milling treatment was performed with a Pulverisette 7 premium line, Fritsch Co., Ltd.

(2) Beads-milling

Each of the powdery Si samples was mixed with zirconia beads (*d* = 0.5 mm) and a solvent (pure water or isopropanol), and the mixture was dealt with by a beads-milling apparatus (LMZ015, Ashizawa Fintech Ltd.).

After either of the above milling processes, the mixture obtained was sieved to separate the sample from the zirconia beads. Then the sample was washed with 1 wt% HF solution to remove a surface SiO_2 layer.

Carbon-coating of Si(sd)-be(ipa)

First, Si(sd)-be(ipa) was placed in a quartz reactor and heat-treated at 1000 °C for 2 h under H_2 flow to reduce a surface oxide layer. Then the temperature was reduced to 800 °C, and CVD was performed for 1 h by using propylene (1 vol% in Ar) as a carbon source.

Assembling coin cells

Method I: The sample (active material) was mixed with conductive additive (Denka black (DB), Denki Kagaku Kogyo Co., Ltd.) and binder polymers (carboxymethylcellulose (CMC), DN-10L, Daicel Fine Chem Ltd.; and styrene butadiene rubber (SBR) (TRD2001, JSR Corporation) dissolved in water) to prepare a slurry. The weight ratio is sample:DB:CMC:SBR = 67:11:13:9. The resulting slurry was pasted on a copper foil and dried at 80 °C for 1 h. Then, the foil was cut into circular shape (*d* = 16 mm) to prepare a working electrode. After it was dried at 120 °C for 6 h under vacuum, a 2032-type coin cell was assembled together with a Li foil (counter electrode) and a polypropylene separator in an Ar-filled glove box. The electrolyte was 1M $LiPF_6$ in a mixture of ethylene carbonate and diethyl carbonate (1:1 by volume ratio).

Method II: Poly(acrylic acid) (Sigma-Aldrich Co., average molecular weight 1,250,000) was mixed with water by using a planetary mixer (ARE-250, Thinky Co., Ltd.) for 15 min at the ‘mixing mode’ and for 15 min at the ‘defoaming mode’ to prepare a homogeneous polymer solution. 1 M NaOH solution (Wako Co. Ltd.) was then added to the solution and the mixture solution was again dealt with by the planetary mixer for 30 min to neutralize 80% of poly(acrylic acid). The resulting homogeneous solution was mixed with an active material and DB to prepare a slurry. The weight ratio of these components was 80:10:10. The following procedure is the same as the case in the Method I.

Method III: Viscous solution of 20 wt% polyamic acid in N-methylpyrrolidone (NMP; U-varnish-A, Ube Industries, Ltd.) was diluted by NMP to prepare a 4.0 wt% polyamic acid solution. Then, an active material, DB, and the polyamic acid solution were mixed by using a mortar and the planetary mixer to prepare a homogeneous slurry, and it was pasted on a copper foil. After drying the foil at 80 °C for 1 h under air, the polyamic acid was imidized at 350 °C for 30 min in N_2. Then the foil was cut into circular shape (d = 16 mm) to prepare a working electrode. The weight ratio of the active material, DB, and polyimide was 75:10:15. A coin cell was assembled basically with the same manner as that of the Method I, except the addition of 2 wt% of vinylene carbonate and 10 wt% of fluoroethylene carbonate into the electrolyte solution.

Method IV: An active material, conductive additive (SUPER C65, TIMCAL Ltd.) and carboxymethyl cellulose (DS = 0.7, Mw = 90,000) were mixed with a buffer solution of pH = 3 (KOH + citric acid) by using a mortar and the planetary mixer to prepare a homogeneous slurry. After the slurry was pasted on a copper foil, it was dried at 80 °C for 1 h under air, and at 100 °C for 2 h under vacuum. Then, the foil was cut into circular shape (d = 16 mm) to prepare a working electrode. The weight ratio of the active material, the conductive additive, and CMC was 80:12:8. The following procedure is the same as the case in the Method III.

Direct observation of the sample after charge/discharge cycling by TEM

For this experiment, coin cells were separately assembled by the Method I, and they were charged/discharged for designed cycles. After then, the working electrodes were taken out from the cells and washed with diethyl carbonate to remove $LiPF_6$, and the active materials were observed by TEM.

S2. SEM and EDX results

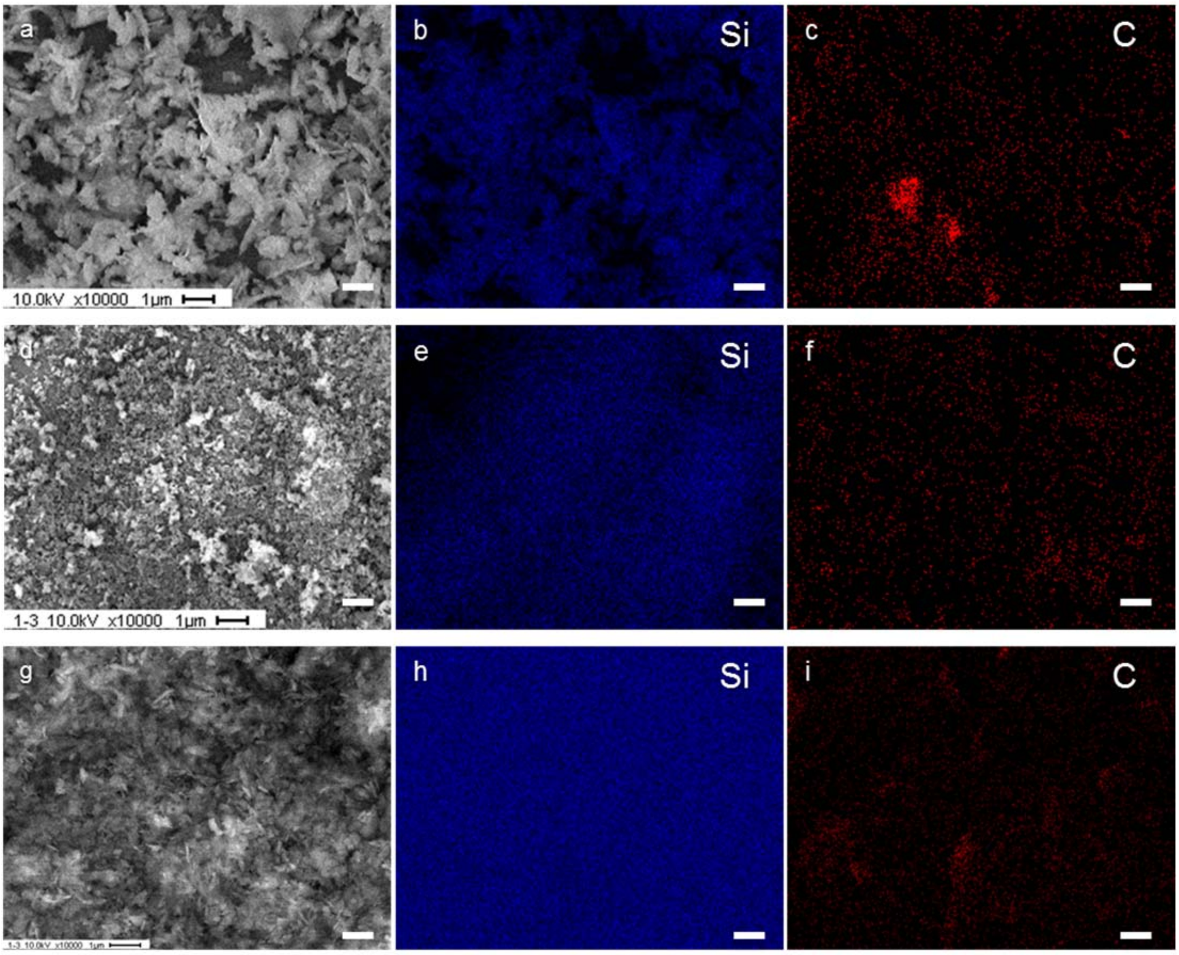

Figure S1. (a,d,g) SEM images and (b,c,e,f,h,i) elemental mappings for (a,b,c) Si(sd), (d,e,f) Si(sd)-ba(w), and (g,h,i) Si(sd)-be(ipa). Scale bars indicate 1 μm.

S3. SEM image of Si(com)-be(ipa) and Si(com)-ba(ipa)

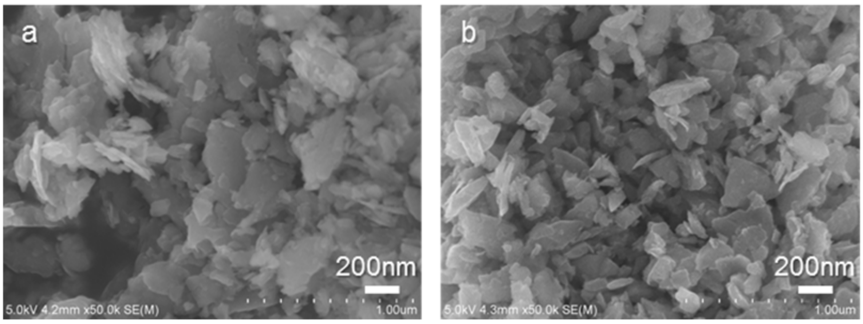

Figure S2. SEM images of (a) Si(com)-be(ipa) and (b) Si(com)-ba(ipa).

S4. Photograph of the mixture of Si(com)-ba(w)/graphite

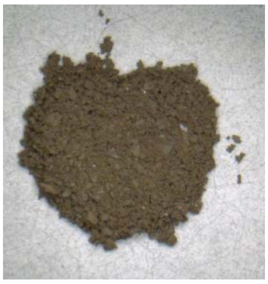

Figure S3. Photograph of the mixture of Si(com)-ba(w) and 5 wt% of graphite (200 mesh, 99.9995% graphite powder, Alfa Aesar).

S5. Crystallite sizes of Si samples

Table S1. Crystallite sizes calculated in each of Si lattice planes for Si(sd), Si(sd)-ba(w) and Si(sd)-be(ipa).

	Lattice plane (nm)				
	(111)	(220)	(311)	(400)	(331)
Si(sd)	85	61	42	63	45
Si(sd)-ba(w)	35	18	15	17	24
Si(sd)-be(ipa)	26	21	17	22	19

S6. Charge/discharge curves of Si(com)-ba(w), Si(sd)-ba(w), Si(sd)-be(ipa), and Si(sd)-be(ipa)/C

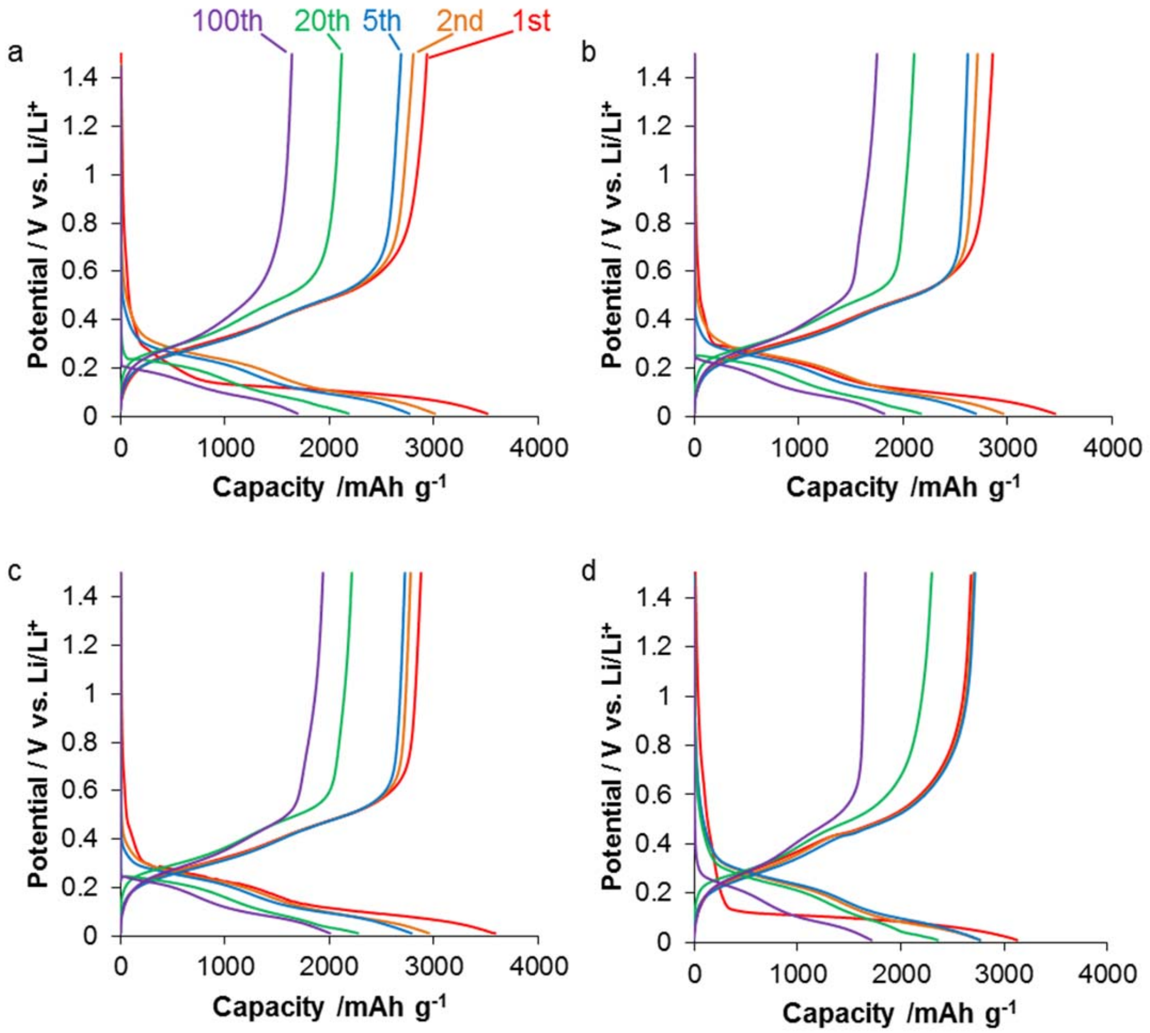

Figure S4. Charge/discharge curves of (a) Si(com)-ba(w), (b) Si(sd)-ba(w), (c) Si(sd)-be(ipa), and (d) Si(sd)-be(ipa)/C without capacity restriction.

S7. Charge/discharge performance of Si(com)-be(ipa) and Si(sd)-be(ipa)

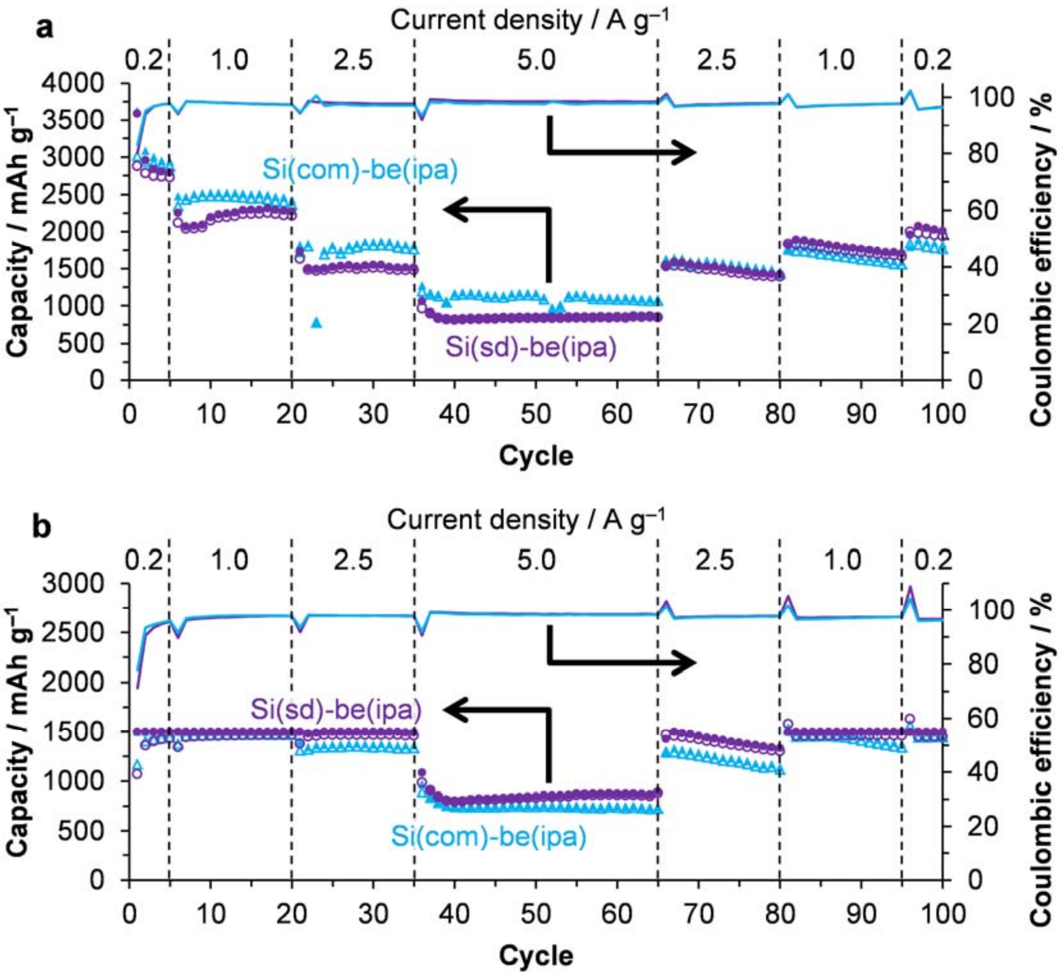

Figure S5. Charge/discharge capacities (open/solid symbols) and coulombic efficiency (solid lines) vs. cycle number (a) without and (b) with capacity restriction up to 1500 mAh g^{-1} measured in 1 M $LiPF_6$ in a mixture of ethylene carbonate and diethyl carbonate (1:1 by volume).
